# DNA2 mutation causing multisystemic disorder with impaired mitochondrial DNA maintenance

**DOI:** 10.1038/s10038-022-01075-4

**Published:** 2022-09-05

**Authors:** Jiayu Sun, Wenwen Su, Jianwen Deng, Yao Qin, Zhaoxia Wang, Yuhe Liu

**Affiliations:** 1 Department of Otolaryngology, Head and Neck Surgery, Peking University First Hospital, Beijing, China; 2 Department of Otolaryngology, Head and Neck Surgery, Beijing Friendship Hospital, Capital Medical University, Beijing, China; 3 Department of Otolaryngology, Head and Neck Surgery, Shanghai Ninth People’s Hospital, Shanghai JiaoTong University School of Medicine, Shanghai, China; 4 Department of Neurology, Peking University First Hospital, Beijing, China

**Keywords:** Disease genetics, Neurological disorders

## Abstract

**Purpose:**

To describe a novel DNA2 variant contributing to defects in mtDNA maintenance and mtDNA depletion syndrome (MDS), and the clinical and histological findings associated with this variation.

**Methods:**

Herein, we describe the case of a patient who presented with hearing loss and myopathy, given the family history of similar findings in the father, was evaluated by sequencing of the deafness gene panel, mitochondrial genome, and the exome. Furthermore, tissue staining, mtDNA copy number detection, mtDNA sequencing, and long-range polymerase chain reaction tests were also conducted on the muscle biopsy specimen. In vitro experiments, including analyses of the mtDNA copy number; levels of ATP, ATPase, and reactive oxygen species (ROS); and the membrane potential, were performed.

**Results:**

The *DNA2* heterozygous truncating variant c. 2368C > T (p.Q790X) was identified and verified as the cause of an mtDNA copy number decrement in both functional experiments and muscle tissue analyses. These changes were accompanied by reductions in ATP, ATPase, and ROS levels.

**Conclusion:**

The *DNA2* variant was a likely cause of MDS in this patient. These findings expand the mutational spectrum of MDS and improve our understanding of the functions of *DNA2* by revealing its novel role in mtDNA maintenance.

## Introduction

Mitochondrial disease (MTD) is a general term for a group of heterogeneous diseases occurring due to mutations of genes related to mitochondrial function [1]. Primary mitochondrial disease can be secondary to both nuclear and mitochondrial gene mutations, and currently, more than such 350 genes have been identified [2]. Such variation and interactions between genes and the environment have been implicated in various mitochondrial diseases (https://www.mitomap.org/MITOMAP). MtDNA maintenance defects are a group of diseases classified under MTD, with mtDNA replication machinery, nucleotide metabolism, and mitochondrial dynamics being most commonly affected due to qualitative (mtDNA deletion) and/or quantitative (mtDNA depletion) abnormalities [3].

MtDNA depletion syndrome (MDS) [4] is one of the mtDNA maintenance defects affecting mtDNA quantity in specific tissues due to nuclear gene mutations. As reviewed [4], mtDNA homeostasis is altered in the following circumstances: (i) defects in genes encoding for members of mtDNA replication and repair machinery, including *POLG* and *TWNK* that affect the minimum replisome and *MGME1* and *RNASEH1* that affect the repair pathways. (ii) defects in proteins involved in mitochondrial dNTPs supply, including *TK2, DGUOK, RRM2B, TYMP, SLC25A4*, and *MPV17*. (iii) defects in proteins involved in mtDNA dynamics, including *OPA1* and *MFN2*. The aforementioned genes can cause both mtDNA deletion and depletion even in the same patient. DNA replication helicase/nuclease 2 (*DNA2*) is a member of the helicase/nuclease family and regulates mtDNA maintenance.

Ronchi et al. reported that *DNA2* (OMIM:601810) variation accounts for approximately 2.7% of mtDNA maintenance disorders in his cohort [5, 6]. The reported pathogenic variants are shown in Table 1. *DNA2* participates in mtDNA replication machinery; however, alterations in *DNA2* have only been described as a cause of multiple mtDNA deletion in skeletal muscle.

**Table 1:** Reported *DNA2* variants and related phenotypes

Variant	NM	Amino acid alteration	Zygosity status	Phenotype	Reference
c.2368C > T	NM_001080449.2	p.Q790X	Heterozygous	Profound sensorineural hearing loss and muscle weakness	Present study
c.1764-38_1764-37 ins (53)	NM_001080449.2	p.Ser588Argfs*4	Homozygous	Microcephalic primordial dwarfism	Tarnauskaitė (2019) [28]
c.74 + 4A > C	NM_001080449.2		Homozygous	Microcephalic primordial dwarfism	Tarnauskaitė (2019) [28]
c.1963A > G	NM_001080449.2	p.Thr655Ala	Heterozygous	Microcephalic primordial dwarfism	Tarnauskaitė (2019) [28]
c.1919C > T	NM_001080449.2	p.Ser640Leu	Heterozygous	Ptosis, arterial hypertension, essential tremor, cataract, and diabetes	Ronchi (2019) [6]
c.2867G > A	NM_001080449.2	p.Arg956His	Heterozygous	Progressive limb-girdle muscle weakness, lower limb hypotonia, and exercise intolerance	Ronchi (2019) [6]
c.1655C > T	NM_001080449.2	p.Ser552Leu	Heterozygous	Ptosis	Ronchi (2019) [6]
c.662C > G	NM_001080449.2	p.Ala221Gly	Heterozygous	Remitting multiple sclerosis and ptosis	Ronchi (2019) [6]
c.2346delT		p.Phe782Leufs*3	Heterozygous	Myopathy associated with ptosis, velopharyngeal weakness, and cardiac involvement	Angel (2019) [21]
c.578T > C		p.Leu193Ser	Heterozygous	Rhabdomyolysis unmasking a mitochondrial disease characterized by a sensorineural hearing loss, ptosis, and lipomas	Angel (2019) [21]
c.1703delA		p.Asn568Ilefs*4	Heterozygous	Congenital onset myopathy and ptosis	Phowthongkum (2017) [19]
c.1888C > T	NM_001080449.2	p.Gln630X	Heterozygous	Multiple congenital joint contractures and hypotonia	Chae (2015) [27]
c.3114 + 6delC (c.3372 + 6delC)	NM_001080449.2	p.Val1065Ilefs*23	Homozygous	Seckel syndrome	Shaheen (2014) [26]
c.851G > A	NM_001080449.1	p.Arg284His	Heterozygous	Decreased facial expressions, moderate diffuse muscle atrophy, relatively mild muscle weakness, an anserine gait with dorsolumbar hyperlordosis, and Gowers’ sign	Ronchi (2013) [5]
c.937A > G		p.Lys313Glu	Heterozygous	Ptosis and progressive myopathy with occasional exertional dyspnea	Ronchi (2013) [5]
c.2167G > A		p.Val723Ile	Heterozygous	Ptosis and progressive myopathy with occasional exertional dyspnea	Ronchi (2013) [5]

In mammals *DNA2* plays essential roles in DNA replication [7, 8], DNA repair [9, 10], the maintenance of genomic stability [11–13], and cell survival and embryonic development [11, 14, 15]. DNA2 co-localizes with Twinkle, a 5′–3′helicase expressed by *TWNK*, unwinding double-stranded mtDNA at the replication fork and facilitating mtDNA synthesis [7, 8]. DNA2 helicase activates and interacts with mtDNA pol γ (encoded by *POLG*), which is the only known mtDNA polymerase and contributes to efficient mtDNA replication by facilitating primer extension [7, 8]. DNA2 and MGME1 both belong to the enzymatic DNA repair system and are involved in mtDNA base excision repair pathways as well as in the removal of RNA primers during replication [16].

Although genes that are functionally related to and/or similar to *DNA2* have been implicated in MDS, *DNA2* variants have not been reported as the cause of MDS to date. In this study, we describe a novel truncating variant of *DNA2* in a 27-year-old male with hearing loss and myopathy. Strikingly, our results revealed that this variant could lead to mtDNA depletion both in vitro and in patient muscle samples.

## Materials and Methods

### Clinical presentation

A 27-year-old male patient presented with a 20-year history of bilateral progressive hearing loss and childhood-onset, gradually aggravating myopathy. He had motor retardation and dyskinesia with a waddling gait and had been unable to walk fast or climb stairs independently since childhood.

**Fig. 1:**
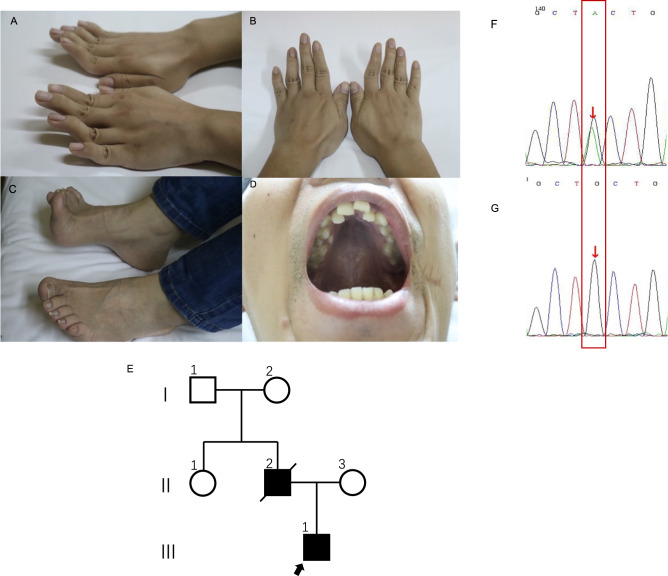
Clinical manifestations, family information, and Sanger sequencing results. **A**, **B** Bilateral excessive bending of the fingers and toes; **C** pes cavus. **D** high arch of the hard palate and irregular tooth arrangement. **E** Family pedigree; the father had the same phenotype, but a genetic analysis was not performed. **F** Patient heterozygous *DNA2* variant verified via Sanger sequencing. **G** A Normative control of the gene

On physical examination, eye movements were bilaterally normal, and pupils were round, regular, and reactive to light. No ptosis was observed. There was no deviation of the tongue or the angle of the mouth. Neck resistance was absent, and the limbs could move freely. The muscle strength of the limbs was near-normal. Tendon reflex of both upper limbs was symmetrically elicited. Knee reflex was absent in both lower limbs. The Achilles tendon reflex of both lower limbs was symmetrically elicited. Rossolimo’s sign, Hoffmann’s sign, and Babinski’s sign were all negative. However, he had a high-arched palate and irregular arrangement of teeth, bilateral excessive bending of the extremities, and pes cavus deformity (Fig. 1A–D). He had never suffered from acidosis or hypoglycemia, with both fasting blood sugars and blood CO_2_ being in the normal range.

His father also complained of bilateral hearing loss and muscular weakness, and had a high-arched palate, irregular arrangement of teeth, bilateral excessive bending of the extremities, and pes cavus deformity. His father had already died of a heart attack at the age of 53; therefore, further physical examination or genetic analysis could not be performed. His mother was healthy with a normal auditory and motor system since childhood, without abnormal physical signs as her son. Her physical and neurological examinations were normal. The course of pregnancy and delivery were both normal (see pedigree in Fig. 1E).

Our patient was identified as the proband, with routine audiology tests revealing bilateral profound sensorineural hearing loss with an unaided pure tone threshold (average of 250 Hz to 8000 Hz) of around 85 dB hearing loss. Radiographic examinations excluded anatomical abnormalities, and cardiac and liver function tests ruled out any cardiac or hepatic diseases when he was received into the hospital before the surgery. We performed bilateral cochlear implantation. Unexpectedly, the patient experienced a generalized tonic-clonic seizure in the postoperative period, accompanied by elevated creatine kinase levels (484 IU/L) lasting for at least 1 month. Seizures did not occur thereafter. One month after the surgery, we turned on the bilateral cochlear, and auditory recovery was satisfactory.

### Genetic analysis

Genomic DNA was extracted from both peripheral blood leukocytes and muscle samples and analyzed via next-generation sequencing (NGS). First, we performed deafness gene detection with GenCap® Deafness Gene Capture Probe V4.0 (MyGenostics, Beijing, China) to search for target genes with the blood sample. Further, to exclude the possibility of other gene variants, we performed exon sequencing with GenCap® Whole Exon Gene Capture Probe V4.0 (MyGenostics, Beijing, China) with the blood sample, and mtDNA sequencing with GenCap® Mitochondrial Loop Gene Capture Probe V1.0 (MyGenostics, Beijing, China) using both blood and muscle samples. MtDNA sequencing analysis was performed with the latest Cambridge version of the mitochondrial genome (rCRS NC_012920) as the reference genome. Electrophoresis of mtDNA (from the muscle sample) was also performed to exclude large-scale mtDNA deletions and rearrangements. The classification of variants followed the American College of Medical Genetics guidelines

After identifying the pathogenic gene (*DNA2*), Sanger sequencing was conducted to confirm the variant with the blood sample. PCR amplification was carried out using the following primers (5′-3′): DNA2-F: AATAAGCTTTCACTCATGCCAAG; DNA2-R: 142 AAGGATTCCTGATGCCATAGAAC. We used the Mutation Surveyor® software to compare the reference sequence with our sequencing data. The classification of variants followed the American College of Medical Genetics guidelines.

### Muscle biopsy and mtDNA copy number detection

Biceps brachii biopsy and mtDNA copy number detection, muscle staining, and ultrastructural studies were performed to verify the outcome of functional assays, as described below. Muscle mtDNA was extracted, and quantitative PCR was performed to determine mtDNA copy number [17]. Fluorescently labeled primers were used to detect the related mitochondrial genome fragments and nuclear genes, so as to calculate the number of mtDNA copies [17]. Western blot of DNA2 in the muscle sample was also performed with the antibody anti-DNA2(Affinity, Cincinnati, OH, USA; DF9453) and anti-GADPH (Proteintech, Rosemont, IL, USA; 10494-1-AP) to ensure successful translation and detect the concentration of DNA2.

### Muscle staining and ultrastructural studies

Muscle tissue samples were stained for histological examinations. Hematoxylin-Eosin (H/E), Modified Gomori trichrome (MGT), Periodic Acid-Schiff (PAS), and Oil Red O (ORO) staining were performed to visualize the morphology of muscle fibers, blood vessels, and connective tissue. Enzyme histochemical analyses of ATPase reactions, and NADH-TR, COX-SDH, and NSE staining were performed to evaluate enzymatic function. Immunohistochemical staining was performed for various proteins associated with muscular dystrophy (i.e., Dystrophin-N, Dystrophin-C, Dystrophin-R, α-Sarcoglycan, β-Sarcoglycan, γ-Sarcoglycan, Dysferlin, and Desmin) and inflammatory myopathy (i.e., CD3, CD4, CD8, CD20, CD68, and MHC-I). Muscle ultrastructure was examined via electron microscopy as per routine methods.

### Functional tests in vitro

Functional experiments, including the analyses of mtDNA copy number; levels of ATP, ATPase, reactive oxygen species (ROS); and membrane potential (MMP), were all performed in triplicate.

#### Plasmid construction

The target gene fragment was prepared using the following primer sequences (5′–3′): *DNA2*-F: CTTGGTACCGAGCTCGGATCCatggagcagctgaacgaact; *DNA2*-R AACGGGCCCTCTAGACTCGAGttattctctttgaaagtcaccca. The target gene was amplified via PCR, and the PCR products were cloned into the pcDNA3.1-3flag-N vector.

#### Cell culture and transfection

HEK293T cells were incubated in Dulbecco’s Modified Eagle Medium containing 10% fetal bovine serum at 37 °C in 5% CO_2_. The cells were separated into four groups, as follows: HEK293T cell control group (Control group), HEK293T cells with empty vector (Empty group), HEK293T cells with 3xFlag-hDNA2 (Wild-type group), and HEK293T cells with 3xFlag-hDNA2 Q790X (Variant group). For transient transfection, cells were cultured for 24 h in an incubator at 37 °C and 5% CO_2_. Thereafter, 7.5 μL of Lipo3000 and 2.5 μg of 3xFlag-hDNA2 plasmid (3xFlag-hDNA2 Q790X plasmid and empty vector for the other groups) with 5 μL of P3000 were separately added to 125 μL of Opti-MEM and mixed at a ratio of 1:1. After 15 min, the compound was added to the medium and incubated for 48 h.

#### Western blotting

Transfected HEK293T cells were washed in phosphate-buffered saline (PBS) and lysed in RIPA buffer (Beyotime, Shanghai, China; P0013B) with protease and phosphatase inhibitors, followed by sonication and centrifugation for 20 min at 4 °C. The purity of recombinant proteins was evaluated via sodium dodecyl-sulfate polyacrylamide gel electrophoresis (SDS-PAGE). Western blotting was performed using whole-cell lysates with the following antibodies: FLAG-TAG (Affinity, Cincinnati, OH, USA; T0003) and anti-GAPDH (Proteintech, Rosemont, IL, USA; 10494-1-AP).

#### mtDNA sequencing analysis in vitro

The mtDNA of transfected HEK293T cells was extracted and subjected to sequencing via the same procedure as for patient blood samples.

#### mtDNA copy number detection

The Human Mitochondrial DNA (mtDNA) Monitoring Primer Set (Takara, Kusatsu, Japan; 7246) was used to quantify the relative human mtDNA copy number via quantitative real-time PCR (RT-qPCR), using nuclear DNA (nDNA) content as a standard. Two pairs of primers (ND1/SLCO2B1 and ND5/SERPINA1) were used for the detection of nDNA and mtDNA, respectively. The Universal Genomic DNA Kit (Kangwei, Shenzhen, China; CW2298) was used to extract sample DNA as per the manufacturer’s instructions. Primer mixes were added, and two mtDNA genes and two nDNA genes were evaluated on the Roche LightCycler480. DNA was amplified using the 2X Color SYBR Green qPCR Master Mix (EZ Bioscience, Roseville, MN, USA; Cat. No. A0012-R2). The threshold cycle (Ct) value for each template was detected via StepOne^**TM**^ Real-Time PCR Fluorescence Quantitative PCR. The differences in Ct values (ΔCt) for the ND1/SLCO2B1 pair (ΔCt1) and the ND5/SERPINA1 pair (ΔCt2) were determined. The 2^ΔCt^ method was used to obtain the final mtDNA copy number.

#### ATP concentration

The ATP concentration was evaluated using the ATP Assay Kit (Nanjing Jiancheng Bioengineering Institute; A095-1-1) as per the manufacturer’s instructions. Briefly, 300 μL of ddH_2_O was added to cells in each treatment group. The cells were ultrasonicated in a hot water bath and vortexed for 1 min. Appropriate reagents were added as per manufacturer instructions, and the samples were maintained at 25 °C for 5 min, and then detected using a microplate reader (Thermo, Waltham, MA, USA; Multiskan GO). The ATP concentration was calculated according to the following formula:

$${{{{{{{\mathrm{ATP}}}}}}}}\,{{{{{{{\mathrm{concentration}}}}}}}}\,( {{\upmu} {\rm {mol}}}/{{{{{{{\mathrm{g}}}}}}}}\,{{{{{{{\mathrm{prot}}}}}}}} ) = 	\, ({{{{{{{{\mathrm{A}}}}}}}}_{{{{{{{{\mathrm{tested}}}}}}}}}-{{{{{{{\mathrm{A}}}}}}_{{{{{\mathrm{control}}}}}}}}})/({{{{{{{{\mathrm{A}}}}}}}}_{{{{{{{{\mathrm{standard}}}}}}}}} - {{{{{{{\mathrm{A}}}}}}}}_{{{{{{{{\mathrm{empty}}}}}}}}}}) \\ 	\times {{{{{{{\mathrm{C}}}}}}}}_{{{{{{{{\mathrm{standard}}}}}}}}} \, \times \, {{{{{{{\mathrm{N}}}}}}}}/{{{{{{{\mathrm{Cpr}}}}}}}}$$

(where C standard represents the Standard concentration of 1 × 10^3^ μmol/L, N indicates the dilution before sample dilution; Cpr indicates the Sample protein concentration in mg/mL.)

#### ATPase concentration

The ATPase concentration was evaluated using the ATPase Assay Kit (Nanjing Jiancheng Bioengineering Institute A016-1) as per the manufacturer’s instructions. Cells were broken via ultrasound treatment in an ice bath (power 20%, ultrasound 5 s, interval 10 s, repeated three times). Cells were then centrifugated at 1000 rpm/min and 4 ˚C for 5 min. The supernatant was obtained for a bicinchoninic acid (BCA) assay. After the enzymatic reaction and phosphorus measurements were completed, samples were detected using a microplate reader. (Thermo Multiskan GO). The detected ATPases included NaK-ATPase, Mg-ATPase, Ca-ATPase, and CaMg-ATPase, and concentrations were obtained based on the following formula:

$$\begin{array}{l}{{{{{{{\mathrm{ATPase}}}}}}}}\,{{{{{{{\mathrm{concentration}}}}}}}}\,\left( {{{{{{{{\mathrm{U}}}}}}}}/{{{{{{{\mathrm{mg}}}}}}}}\,{{{{{{{\mathrm{prot}}}}}}}}} \right) = \left( {{{{{{{{\mathrm{measured}}}}}}}}\,{{{{{{{\mathrm{OD}}}}}}}} - {{{{{{{\mathrm{controls}}}}}}}}\,{{{{{{{\mathrm{OD}}}}}}}}} \right)/{{{{{{{\mathrm{standard}}}}}}}}\,{{{{{{{\mathrm{OD}}}}}}}}\\ \times {{{{{{{\mathrm{Standard}}}}}}}}\,{{{{{{{\mathrm{concentration}}}}}}}}\left( {1\,\upmu {{{{{{{\mathrm{mol}}}}}}}}/{{{{{{{\mathrm{mL}}}}}}}}} \right) \times {{{{{{{\mathrm{Dilution}}}}}}}}\,{{{{{{{\mathrm{of}}}}}}}}\,{{{{{{{\mathrm{sample}}}}}}}}\,{{{{{{{\mathrm{in}}}}}}}}\,{{{{{{{\mathrm{reaction}}}}}}}}\,{{{{{{{\mathrm{system}}}}}}}} \\ \times 6/{{{{{{{\mathrm{Protein}}}}}}}}\,{{{{{{{\mathrm{concentration}}}}}}}}\,{{{{{{{\mathrm{of}}}}}}}}\,{{{{{{{\mathrm{the}}}}}}}}\,{{{{{{{\mathrm{sample}}}}}}}}\,{{{{{{{\mathrm{to}}}}}}}}\,{{{{{{{\mathrm{be}}}}}}}}\,{{{{{{{\mathrm{tested}}}}}}}}\,\left( {{{{{{{{\mathrm{mg}}}}}}}}\,{{{{{{{\mathrm{prot}}}}}}}}/{{{{{{{\mathrm{mL}}}}}}}}} \right)\end{array}$$

#### ROS detection via 2′-7′dichlorofluorescin diacetate (DCFH-DA)

Cells were washed with PBS. DCFH-DA was diluted to 10 μM/L. The collected cells were added to DCFH-DA and incubated at 37 °C and 5% CO_2_ for 20 min. The cells were washed with PBS three times to remove the free probe and were then resuspended in PBS after centrifugation. ROS were immediately detected using a flow cytometer (BD CytoFLEX S), and the results were analyzed using FlowJo™ Software.

#### MMP with JC-1

JC-1 working solution was added to cells for incubation at 37 °C. During incubation, an appropriate volume of JC-1 buffer, 1 mL of staining buffer (5×), and 4 mL of ultrapure water were added. The free probe was removed after incubation. A flow cytometer (BD CytoFLEX S) was used to detect MMP [18], and the results were analyzed using FlowJo.

### Statistical analysis

Data are presented as the mean ± SD and were analyzed using analysis of variance. SPSS 25.0 (IBM Corp., Armonk, NY, USA) was used for all statistical analyses. A two-tailed *p*-value ≤0.05 indicated a statistically significant difference.

## Results

### Genetic analysis revealed a novel DNA2 variant

A DNA2 heterozygous truncating variant, c. 2368C > T; p.Q790X, in exon 15(NM_001080449.2), was detected by sequencing both the deafness gene panel and exome, resulting in a change from glutamine to a termination codon at amino acid residue 790, as confirmed via Sanger sequencing (Fig. 1F, G). The variant was classified as likely pathogenic based on the American College of Medical Genetics guidelines. To the best of our knowledge, this variant has not been previously reported in the literature or included in the Human Gene Mutation Database or ClinVar. The deafness gene panel also detected other gene variants (i.e., COL1A1, ITM2B), as did exome sequencing (i.e., DMD, DYS) (details are shown in Supplementary Table S1).

mtDNA sequencing from blood and muscle sample, and the reconstructed HEK293T cells, helped excluded the possibility of pathogenic point mutation. Similarly, mtDNA long-range PCR from muscle sample helped excluded large-scale deletions or repetitive variations (the long-range PCR of muscle sample was shown in Supplementary information Fig. S1). Other variants with unknown clinical significance from mtDNA sequencing included m.10398A > G, m.14696A > G, and m.16189T > C.

### mtDNA depletion and biochemical changes confirmed via in vitro functional analyses

**Fig. 2:**
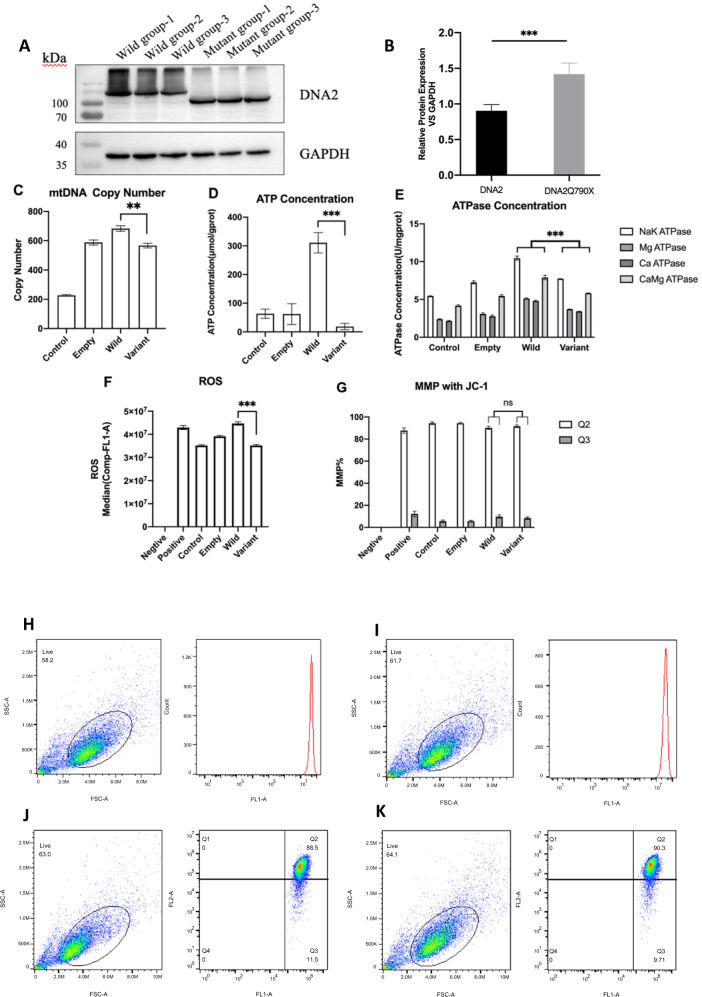
Functional experiments in vitro. All the experiments were repeated for three times. **A**, **B** Western blotting of both the Wild-type group and Variant group, confirming stable expression of the variant. **C** mtDNA copy number for different groups. The copy number was significantly lower in the Variant group than in the Wild-type group (83%). **D** ATP concentration (µmol/gprot). The concentration was lowest in the Variant group. **E** ATPase concentration (µmol/gprot) for different enzymes. ATPase levels tended to be higher in the Wild-type group than in the Mutant group. **F** ROS levels. The Variant group had a significantly lower ROS level than that of the Wild-type group. **G** MMP levels. Differences were not observed among groups. Error bars of one standard deviation were also shown in **C**–**G**. **H**–**K** ROS and MMP levels in the Wild-type group and Mutant group. **H** ROS levels in the Wild-type group; **I** ROS levels in the Variant group; **J** MMP levels in the Wild-type group; **K** MMP levels in the Variant group

Western blotting confirmed the stable expression of the target protein, with a smaller size in the Variant group than in the Wild-type group (Fig. 2A). This was consistent with the results of genetic analysis, confirming that the mutation resulted in a truncated variant. The protein concentration, when standardized against the internal reference protein GAPDH, was higher in the Variant than in the Wild-type group (*p* = 0.007), indicative of a normal transcription and translation process (Fig. 2B). The mtDNA copy numbers in the Control group, Empty group, Wild-type group, and Variant group were 227.18 ± 5.40, 588.16 ± 17.07, 683.18 ± 18.95, and 566.90 ± 15.64, respectively. The copy number was significantly lower in the Variant group than in the Wild-type group (*p* = 0.008). These findings confirmed that the observed *DNA2* variant led to a substantial decrease in mtDNA (Fig. 2C).

ATP concentrations (µmol/gprot) in the Control group, Empty group, Wild-type group, and Variant group were 63.34 ± 16.00, 62.10 ± 36.40, 310.67 ± 35.46, and 18.81 ± 11.02, respectively. The significant difference (*p* < 0.001) between the Wild-type group and Variant group indicated that the variation resulted in a dramatic decrease of ATP concentration (Fig. 2D). The difference in ATPase concentration (µmol/gprot) between the Wild-type and the Variant groups was significant for all four ATPases (Fig. 2F). These results indicated that the variation significantly decreased both ATP and ATPase concentrations, potentially leading to alterations in ionic concentrations and enzymatic reaction speeds.

The difference in ROS levels between the Wild-type and Variant groups was also significant (*p* < 0.0001) (Fig. 2F, H, I), whereas MMP, Q2/Q3, and Q3 did not differ significantly between the groups (Fig. 2G, J, K).

### Verification of variant pathogenicity

Western blot of DNA2 from muscle sample confirmed the stable translation, and the concentration of DNA2 was the same as that seen in the control group (Supplement Material Fig. S2). The mtDNA copy number obtained from the patient’s muscle tissue from the patient was almost two-folds lower than in tissue from healthy age-matched controls (Table 2). This finding, combined with the results of the in vitro mtDNA copy number analysis, further confirmed that the truncating variant could lead to mtDNA depletion.

**Table 2:** mtDNA copy number in muscle samples from the patient and a group of healthy control of the same age group

Copy number	Ratio to relevant age group control	Age group (y)	Mean ± standard deviation of control
1564 ± 41	44.7%	21–40	3495 ± 586

**Fig. 3:**
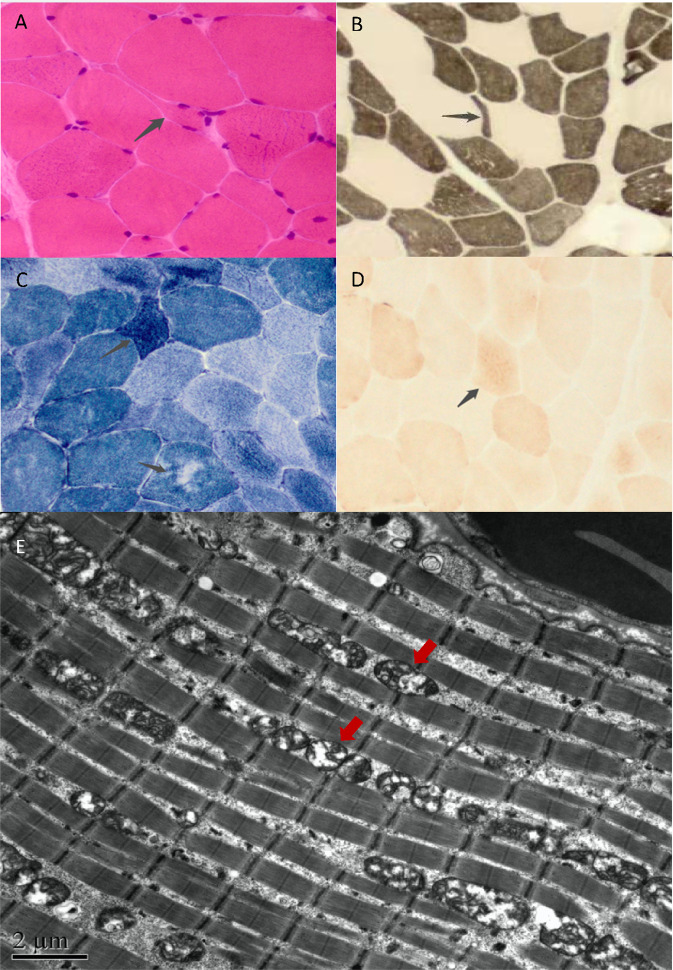
Muscle staining and ultrastructural analyses. **A** HE staining, small angular and round atrophic muscle fibers were scattered. **B** Acid reaction series of ATPase confirmed vacuole formation in muscle fibers, and atrophy of type II muscle fibers could be seen. **C** NADH-TR staining revealed that the distribution of oxidase activity in a few muscle fibers was uneven, and atrophic muscle fibers exhibited intense staining. **D** COX-SDH staining revealed the enzyme activities of many muscle fibers decreased, with granular deep staining in individual muscle fibers. No RBF, SSV, or Cox negative muscle fibers were found. **E** The size and morphology of mitochondria were uneven, and the cristae structure was not clear. The number of mitochondria increased, with an enlarged volume and disrupted cristae

Staining revealed myopathy-like pathological changes, as shown in Fig. 3A–D. COX-SDH staining showed that the enzymatic activities of many muscle fibers had decreased, with granular deep staining noted in individual muscle fibers. No RBF, SSV, or Cox-negative muscle fibers were found. Ultrastructural analysis indicated abnormal mitochondrial morphology with an increase in mitochondrial quantity, enlarged volume, and disrupted cristae, altogether suggestive of mitochondrial dysfunction (Fig. 3E).

## Discussion

### Pathogenesis

We identified a novel variant with a truncation in ATPase/helicase domain of DNA2 [5, 19], more specifically, in Helicase 1A [6], which was expected to lead to a loss of both ATPase and helicase function based on its location and our functional tests. We concluded that the loss of ATPase and helicase function affected mtDNA stability, resulting in a decreased mtDNA copy number and morphological abnormalities of the mitochondria, eventually leading to ATP and ATPase shortage and lower ROS generation. These alterations would have detrimental effects on energy-consuming organs, leading to motor retardation, deafness, and seizures. Thus, we believe that the patient suffers from encephalomyopathy MDS [20].

Previous studies believe that haploinsufficiency was the pathogenic mechanism, as wild-type *DNA2* could not make up for the adverse effect of variation in nuclease activity [5, 21]. We also suspected that haploinsufficiency may have been the disease mechanism in this patient, and that multisystem dysfunction might have arisen from the suboptimal availability of DNA2 protein. However, further functional analyses are needed to confirm the exact underlying mechanism.

From the mtDNA sequencing, we also found point mutations such as m.10398A > G, m.14696A > G, m.16189T > C. Notably, m.10398A > G has been implicated in malignancies [22], while m.16189T > C has been reported to cause diabetes [23]. However, there is lack of evidence in the role of these variants in the incidence of hearing loss or myopathy. m.14696A > G has been reported as a factor leading to progressive encephalopathy [24], and was once reported to cause pediatric onset encephalomyopathy with impaired respiratory chain activity in a girl [25]. Her clinical manifestations included delayed psychomotor development, muscle hypotonia, and severe dysphasia. Histologic examination of the muscle showed ragged red fibers and increased mitochondria, and brain magnetic resonance imaging showed abnormal brain morphology. Our patient exhibited similar early-onset multisystemic dysfunction, but dysphasia and the histological or morphological features noted in the girl were conspicuously absent. To further exclude the pathogenicity of this variant, we firstly confirmed the presence of the variant by Sanger sequencing of blood samples of both the patient and his mother. Subsequently, the heteroplasmy level of this variant was detected with mtDNA using NGS, and found to be 99.89% of the muscle sample of the patient. The heteroplasmy level of the blood sample of the patient was 88%, and that of the mother was 84%. To further rule out its pathogenicity, NGS of mtDNA starting from long-range PCR amplicons of both the patient’s and his mother’s blood samples was also performed. The heteroplasmy level of the patient was found to be 92%, and that of the mother, 95%. Therefore, we concurred that this variant was not the pathogenic factor in this patient, since his mother was asymptomatic even though they had similar heteroplasmy levels (see Supplementary Material for the results of Sanger sequencing).

The deafness gene panel also detected other gene variants (i.e COL1A1, ITM2B), but their pathogenicity was uncertain as per the American College of Medical Genetics guidelines. Furthermore, the main clinical manifestations failed to match that seen in the patient. DMD and DYSF variants were detected from exome sequencing; however, they were excluded based on muscle staining results. Therefore, these gene variants were excluded from consideration (details are shown in Supplementary Table S1).

### *DNA2* variation can cause mtDNA depletion syndrome

Previously reported variants of *DNA2* have mainly harbored point (missense) mutations, involving change in only one amino acid residue, leaving a functionally intact sequence. These variants can also cause various pathologies, such as progressive external ophthalmoplegia (PEO, MIM615156) [5] or Seckel syndrome type 8 (MIM 615807) [26], as described in Table 1. Only one other immature truncating mutation, c.1888C > T (p.Gln630Ter), has been reported to date; this mutation was also reportedly located in the Helicase domain, similar to the location determined in our study. However, analyses of mtDNA copy number and functional tests were not performed in the aforementioned study [27].

The novel truncating variant discovered in our study was associated with decreasing mtDNA quantity and ATPase. To the best of our knowledge, this has never been reported before. Meanwhile, the reserved nuclease activity, conversely, might help retain mtDNA quality. Therefore, no deletion was found in mtDNA. Interestingly, *DNA2*-related deafness has not been previously reported, which might be explained by sufficient energy supplementation in those cases. However, our results suggest that mtDNA depletion and extreme ATP insufficiency might cause severe deafness. The copy number reduction in our patient was lesser than that previously reported in a few cases, and this might be explained, at least in part, by the less substantial loss of DNA2 function in replication.

In conclusion, we reported a novel *DNA2* variant, c. 2368C > T; p.Q790X, associated with deafness and motor dysfunction. We further confirmed a causative link with MDS via mtDNA copy number analyses, functional assays, and staining. mtDNA depletion was accompanied by mitochondrial distortion, ATP and ATPases insufficiency, and decreased ROS levels. The current findings expand on the mutational spectrum of MDS as well as on our understanding of DNA2 as a regulator of mtDNA maintenance. Further research, including investigations into the CGH-array of the patient, biochemical analysis of DNA2 activities in vitro, and animal experiments, are warranted to confirm the pathogenicity of the novel variant and elucidate the mechanism through which it contributes to hearing loss. The patient should undergo further follow-up as well.

## Supplementary information

Supplementary Material

## Data Availability

Data and materials, such as the Sanger sequencing electropherograms, primer sequences, strains, and plasmids are available with the corresponding author upon reasonable request. The authors affirm that all data necessary for confirming the conclusions of the paper are present within the article, figures, and tables.
