# Supplementary material for: DNA2 mutation causing multisystemic disorder with impaired mitochondrial DNA maintenance
Source: J Hum Genet. 2022 Sep 5;67(12):691–9. doi: 10.1038/s10038-022-01075-4 (PMC9691460; doi:10.1038/s10038-022-01075-4)
Supplement: Supplementary file 1 — Supplementary Material [file 10038_2022_1075_MOESM1_ESM.docx]

Supplementary material

Table S1. Other variants of the patient via two gene panels

| Gene | Chromosome position | Transcript exon | Nucleotide and Amino Acid | Homozygous / Heterozygous | Protein function prediction | Pathogenicity analysis |
| --- | --- | --- | --- | --- | --- | --- |
| *DMD* | chrX:31496426-31496431 | NM_004006; exon5 | c.8729_8734delAGGTCAinsTGGTCG  (p.E2910_N2912delinsVVD) | hemi | - | Likely pathogenic |
| *DYSF* | chr2:71744159 | NM_003494; exon9 | c.896G>A (p.G299E) | het | D | Likely pathogenic |
| *DYSF* | chr2:71886111 | NM_003494; exon43 | c.4742G>A (p.R1581H) | het | LD | Uncertain |
| *DYSF* | chr2:71827854 | NM_003494; exon34 | c.3725G>A (p.R1242H) | het | LD | Uncertain |
| *DYSF* | chr2:71740969 | NM_003494; exon6 | c.581C>A (p.P194Q) | het | B | Uncertain |
| *COL1A1* | chr17:48266829 | NM_000088; exon39 | c.2738C>T (p.T913I) | het | D | Uncertain |
| *ITM2B* | chr13:48832958 | NM_021999; exon5 | c.590A>G (p.Y197C) | het | D | Uncertain |

Protein function was predicted with software REVEL, D：Predicted to be harmful；LD：Predicted as potentially harmful；B: Predicted to be benign；-：unknown

8000

5000

10000


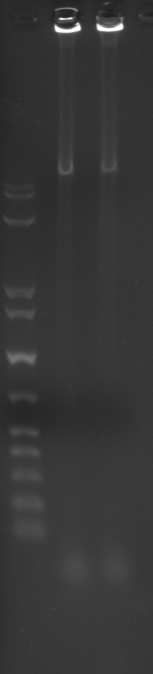


Patient

Control

bp

Figure S1 Long-range PCR of Muscle mtDNA: no large-scale deletion was found in the patient mtDNA extracted from muscle sample.


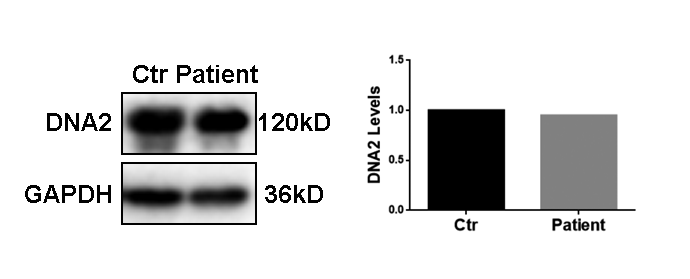


Figure S2 Western blot of DNA2 extracted from muscle sample of the patient, indicating a successful translation of the truncated protein *in vivo*. The concentration of DNA2 in the patient muscle was the same as a healthy control.

Figure S3 Sanger sequencing of m.14696 A>G with the blood sample of the patient and the mother to confirm the variant in the proband and the family member.


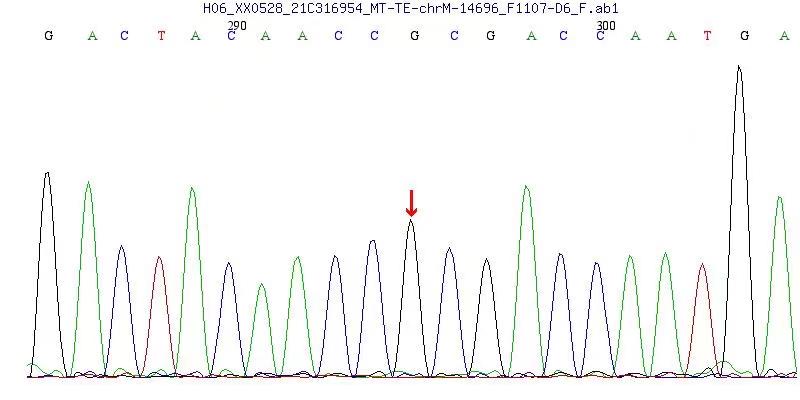

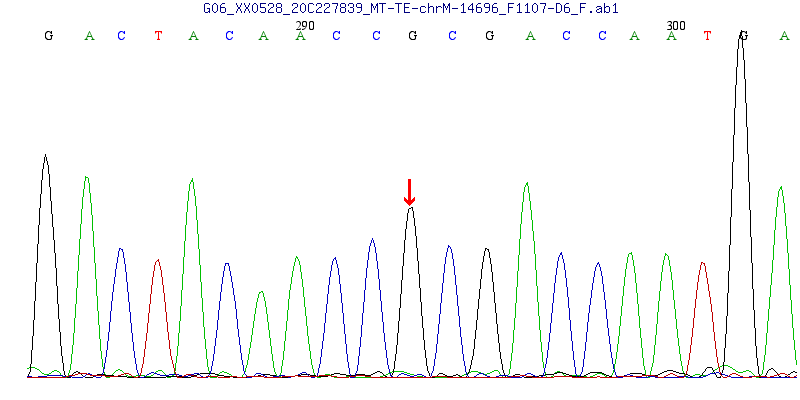


The patient

The mother
